# A new method for noninvasive venous blood oxygen detection

**DOI:** 10.1186/s12938-016-0208-8

**Published:** 2016-07-19

**Authors:** Xu Zhang, Meimei Zhang, Shengkun Zheng, Liqi Wang, Jilun Ye

**Affiliations:** School of Medicine, Shenzhen University, Shenzhen, China; National-Regional Key Technology Engineering Laboratory for Medical Ultrasound, Shenzhen, China; Guangdong Key Laboratory for Biomedical Measurements and Ultrasound Imaging, Shenzhen, China

**Keywords:** Noninvasive method, SvO2, External stimulation signal

## Abstract

**Background:**

Blood oxygen saturation of vein (SvO2) is an important clinical parameter for patient monitoring. However, the existing clinical methods are invasive, expensive, which are also painful for patients.

**Methods:**

Based on light-absorption, this study describes a new noninvasive SvO2 measurement method by using external stimulation signal to generate cyclical fluctuation signal in the vein, which overcomes the low signal-to-noise ratio problem in the measurement process. In this way, the value of SvO2 can be obtained continuously in real time.

**Results:**

The experimental results demonstrate that the method can successfully measure venous oxygen saturation by artificial addition of stimulation. Under hypoxic conditions, the system can reflect the overall decline of venous oxygen saturation better. When the results measured by the new method are compared with those measured by the invasive method, the root mean square error of the difference is 5.31 and the correlation coefficient of the difference is 0.72. The new method can be used to measure SvO2 and evaluate body oxygen consumption, and its accuracy needs improvement.

**Conclusions:**

Real-time and continuous monitoring can be achieved by replacing invasive method with noninvasive method, which provides more comprehensive clinical information in a timely manner and better meet the needs of clinical treatment. However, the accuracy of the new noninvasive SvO2 measurement based on light-absorption has to be further improved.

**Electronic supplementary material:**

The online version of this article (doi:10.1186/s12938-016-0208-8) contains supplementary material, which is available to authorized users.

## Background

SvO2 and SaO2 (blood oxygen saturation of artery) have been established as the basic parameters to evaluate oxygen delivery process and monitor patient’s oxygen consumption. According to Fick Formula in Eq. (1)[1], the oxygen circulation in human body can be analyzed completely only by combining SvO2 and SaO2 together. Therefore, SvO2 has a significant meaning in evaluating the health of patients.

1 $$\text{SvO2 = SaO2} - \text{VO 2} \div \text{(1}\text{.34} \times \text{CO} \times \text{Hb)}$$

On the other hand, there is no existing noninvasive or continuous device for measuring SvO2 in the market, even though light absorption method has already been widely used to evaluate SaO2. Besides the high price and inconvenient, invasive method also has more risk in clinical SvO2 measurement.

Due to the lack of fluctuation signal in vein, SvO2 can’t be measured in the same way as SaO2 is measured, for which light absorption method is valid because of the obvious impulse in artery. To overcome this problem a new noninvasive light-absorption method for SvO2 measurement is proposed. In this method, we innovatively create vein impulse similar to artery impulse by artificially adding external stimulation signal during measurement, which enables the extraction of the stable venous signal from normal arterial pulse signal.

## Methods

According to the Lambert–Beer’s law, the intensity of light through the medium has different degrees of attenuation which depending on the thickness and concentration of the solution. Because the transmission light intensity will decrease with the increasing of the concentration and the thickness of the solution, the absorbance of a single solution can be calculated by Eq. (2)

2 $$A = \ln \left( {\frac{Ii}{It}} \right) = a \cdot C \cdot L$$

where *A* represents absorbance, the natural logarithm of the ratio of the incident light intensity to the transmitted light intensity; *Ii* represents the incident light intensity and *It* represents the intensity of the transmitted light, *a* represents the absorption coefficient, with different values for different solutions; *C* represents the solution concentration; *L* represents the optical path and is the distance that light travels through in the solution [2].

Be similar to mixed solution, the absorbance of blood can be calculated by optical absorption model for mixed solution as shown in Eq. (3), and there is no limit on the number of components in the solution. As we all know, the absorbance of the mixed solution satisfies the superposition theorem and is the superposition of all solution ingredients’ absorbance at different concentrations and different absorption coefficients.

3 $$A = \ln \left( {\frac{Ii}{It}} \right) = \mathop \sum \limits_{k = 1}^{\infty } a_{k} \cdot C_{k} \cdot L$$

Equation (4) is the mixed absorbency at 660 and 940 nm with oxygen free hemoglobin and oxygenated hemoglobin under static condition, where *λ* is the wavelength of the original light.

4 $$A(\lambda ) = \ln \left( {\frac{{I_{i} (\lambda )}}{{I_{t} (\lambda )}}} \right) = a_{Hb} (\lambda ) \cdot aC_{Hb} (\lambda ) \cdot L + a_{HbO2} (\lambda ) \cdot aC_{HbO2} (\lambda ) \cdot L$$

In dynamic state, the intensity change of the transmission light is induced by the change of optical path due to congestion artery, as shown in Eq. (5)

5 $$I\left( {\lambda ,t} \right) = I_{0} (\lambda )exp( - (s\beta_{0} \left( \lambda \right) + (1 - s)\beta_{r} (\lambda ))l(t))$$

where *I*_o_ and I represent the intensity of the incident light and the transmission intensity of the original light respectively. *λ* denotes the wavelength of the original light. *t* is the time. *S* is the oxygen saturation. β_0_ and β_γ_ are the absorption coefficients for the Hb and HbO2 in the solution. *l*(*t*) is the optical path from the light source to the photoelectric sensor [3].

Equation (5) can be easily reformulated as Eq. (6)

6 $$\frac{{ d\left( {\ln I(\lambda ,t)} \right)}}{dt} = - \left( {s\beta_{0} \left( \lambda \right) + \left( {1 - s} \right)\beta_{r} \left( \lambda \right)} \right)\frac{{d\left( {l\left( t \right)} \right)}}{dt}$$

Then Eq. (7) can be derived from Eqs. (4), (5) and (6).

7 $$S = \frac{{\frac{{dlnI\left( {\lambda_{IR} } \right)}}{dt}\beta_{r} \left( {\lambda_{R} } \right) - \frac{{dlnI\left( {\lambda_{R} } \right)}}{dt}\beta_{r} \left( {\lambda_{IR} } \right)}}{{\frac{{dlnI\left( {\lambda_{R} } \right)}}{dt}(\beta_{0} \left( {\lambda_{IR} } \right) - \beta_{r} \left( {\lambda_{IR} } \right)) - \frac{{dlnI\left( {\lambda_{IR} } \right)}}{dt}(\beta_{0} \left( {\lambda_{R} } \right) - \beta_{r} \left( {\lambda_{R} } \right))}}$$

Assuming that Eq. (8) holds.

8 $$\frac{{d\left( {lnI(\lambda ,t)} \right)}}{dt} \cong ln\left(\frac{{I(t_{2} ,\lambda )}}{{I(t_{1} ,\lambda )}}\right)$$

We can rewrite Eq. (7) to Eq. (9) to calculate oxygen saturation

9 $$S = \frac{{\beta_{r} \left( {\lambda_{R} } \right) - R\beta_{r} \left( {\lambda_{IR} } \right)}}{{R(\beta_{0} \left( {\lambda_{IR} } \right) - \beta_{r} \left( {\lambda_{IR} } \right))(\beta_{0} \left( {\lambda_{R} } \right) - \beta_{r} \left( {\lambda_{R} } \right))}}$$

where R (molar extinction coefficient) [4] is defined below and all the other variables are constant.

10 $$R = \frac{{ln(I(t1,\lambda_{R} )/I(t2,\lambda_{R} ))}}{{ln(I(t1,\lambda_{IR} )/I(t2,\lambda_{IR} ))}} \cong \frac{{\frac{{dlnI\left( {\lambda_{R} } \right)}}{dt}}}{{\frac{{dlnI\left( {\lambda_{IR} } \right)}}{dt}}}$$

According to Eqs. (7) and (9), the full parameters of the pulse oxygen saturation can be obtained by calculating the alternating current (AC) and direct current (DC) values of red light and infrared light of signals [5].

Usually, pulse fluctuation can lead venous oxygen signal overlap with the artery blood oxygen signal, which causes problem of low signal (SvO2)-to-noise (SaO2) ratio. Therefore, the extraction of the weak venous blood oxygen signal from artery blood oxygen signal is the key to non-invasive SvO2 measurement. Based on clinical studies, a new method has been proposed. It measures the periodic fluctuation vein signal, whereas a new system has been designed to verify the method.

**Fig. 1:**
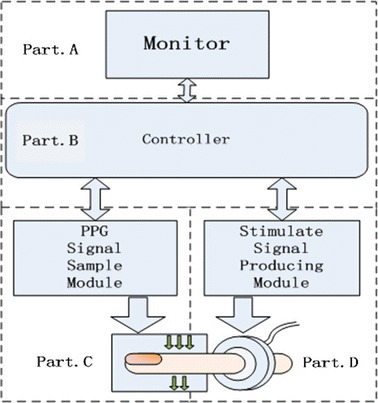
Components of system

As Fig. 1 shows, the system consists of four main parts. Part.A is the monitor for data calculation and waveform display. Part.B is the system controller. This module can control the work of Part.A, Part.C and Part.D. Part.C is the photoplethysmography (PPG) signal sample module getting photoelectric volume pulse wave data. Part.D is the signal generation module. In this part, a ring-shaped inflatable air cuff (Part.D of Fig. 1) is used to produce stable signal as stimulate signal. By controlling Part.D, Part.C can gain the arterial PPG signal and the mixed PPG signal respectively, which is then displayed in Part.A.

**Fig. 2:**
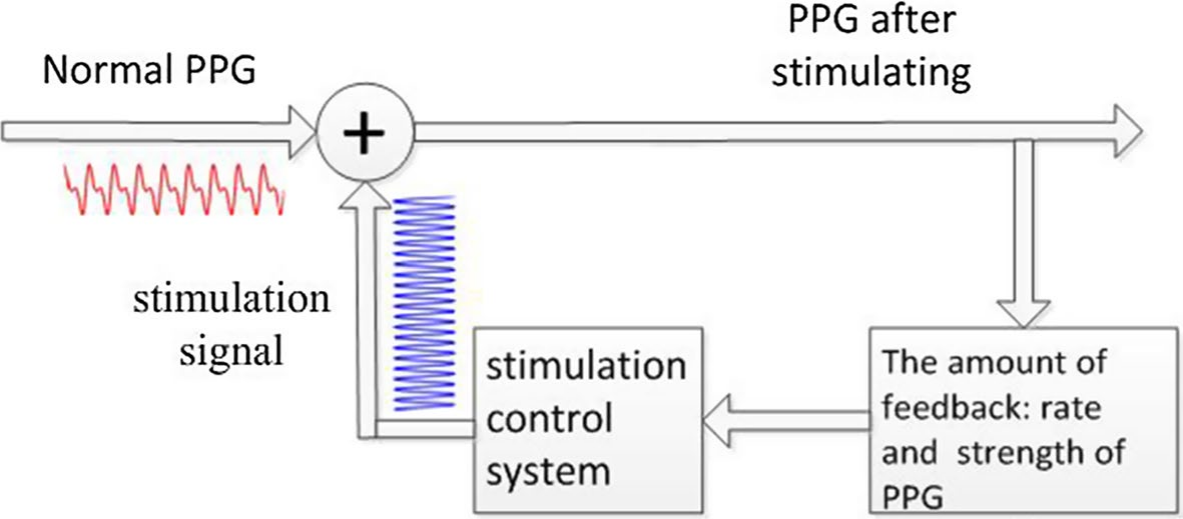
Stimulation feedback system

**Fig. 3:**
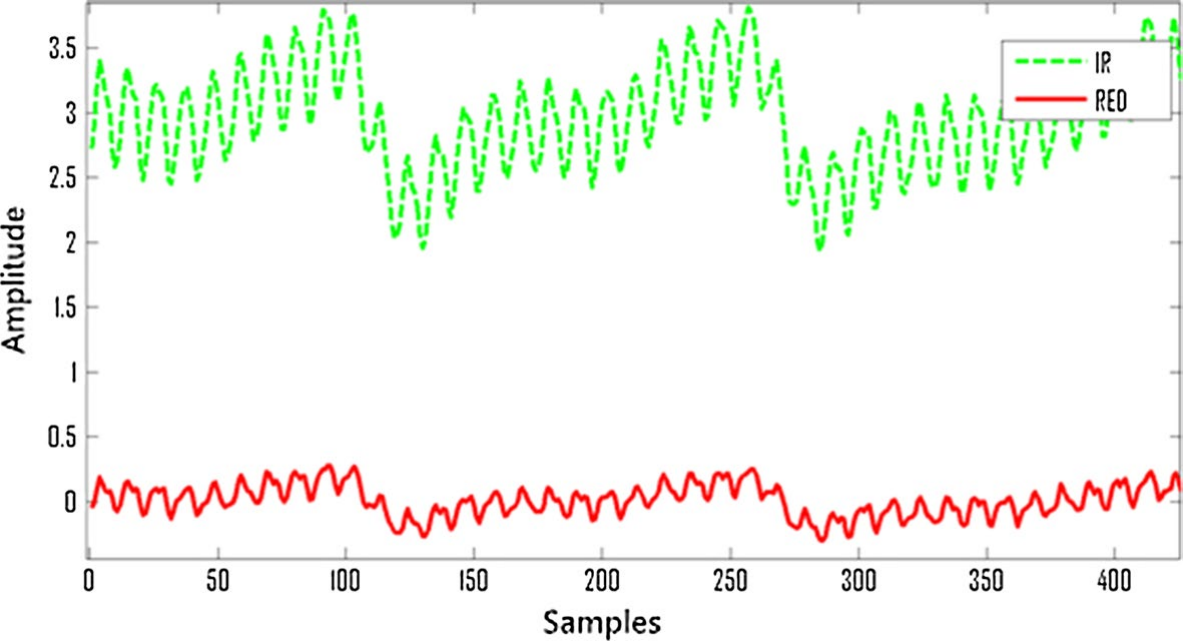
The difference between normal and stimulate signal

Periodic pressure can be added to finger by inflating and deflating the air cuff periodically. NIBP (non-invasive blood pressure) is used as a controllable parameter to set the threshold pressure for different people, while the frequency of inflation and deflation are controlled by a high-precision timer. Figure 2 is the control chart for the venous signal enhancement system. The stimulation control system is the module that controls the venous signal enhancement. The feedback adjustment is controlled by MCU. The detailed process is as follows: firstly, PPG of arteries and veins can be detected by dual-wavelength pulse oxygen measurement system; secondly, the signal frequency and strength of the two kinds of PPG are used as feedback to increase the signal of vein; thirdly, with feedback of the relationship between the signal-to-noise ratio of vein and arterial, the stimulation control system to stay in a stable and acceptable condition can be maintained (the signal frequency of the two kinds of PPG is different). Figure 3 briefly shows the difference between normal signal and stimulated signal. In the signals of IR and RED, every PPG contains multiple excitation signals. Besides, under the stimulation condition, both arterial signal and venous signal are affected.

The SvO2 signal extraction process generally includes three steps. Firstly, the stimulated signal is wiped out and the normal SpO2 signal is calculated. Secondly, the signal which is too far from our stimulation frequency is filtered and the peak of artificially stimulated signal is used as input data to calculate Mixed-SpO2 in the normal way which is same to the calculation of SpO2. Thirdly, SvO2 is calculated. In this paper, venous PPG generated by the stimulation signal is used to calculate the venous oxygen saturation, which is the same as arterial oxygen saturation PPG used to calculate the arterial oxygen saturation. The PPG is recorded at 660, 940 nm for red and infrared transmission intensity changes. In theory, if PPG of the arterial blood signal can be used to calculate the arterial oxygen saturation, PPG of the venous blood signal can also be used to calculate the venous oxygen saturation. Because the features of the two signals are the same, SvO2 can be calculated use the same method to calculate SpO2. In fact, the signal the system acquired is the mixed signal. In the case of ideal signal to noise ratio, venous oxygen saturation can be calculated by formula (11) and formula (12).

11 $${\text{Mixed}}\_{\text{SpO}}2 = ({\text{SpO}}2 + {\text{SvO}}2)/2$$

12 $${\text{R}}\_{\text{SpO}}2 = {\text{R}}\_{\text{SvO}}2 = \left(\frac{{\Delta {\text{I}}_{\text{t}}^{660} }}{{{\text{I}}_{\text{t}}^{660} }}\right)/\left(\frac{{\Delta {\text{I}}_{\text{t}}^{940} }}{{{\text{I}}_{\text{t}}^{940} }}\right)$$

**Fig. 4:**
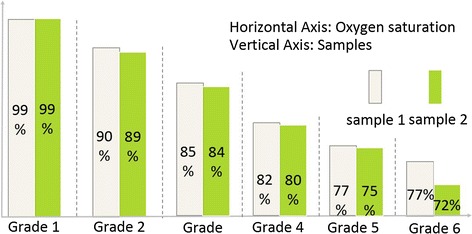
Sampling points

At last, the invasive blood gas analysis is utilized as the golden criterion to calibrate the data measured by our system, referring to the calibration process of SpO2. The blood gas analysis platform of the laboratory can control the oxygen concentrations by adjusting the intake of oxygen. The system records the values of SaO2 and SvO2 in noninvasive method, at the same time the blood samples of artery and vein are sampled in 100–70 % hypoxic state. 24 samples of venous blood were sampled near from our finger in consistent with the process showed in Fig. 4. The sampling was conducted at six oxygen saturation levels, with two samples collected at each level. This was repeated twice to acquire the 24 samples.

## Results and discussion

**Fig. 5:**
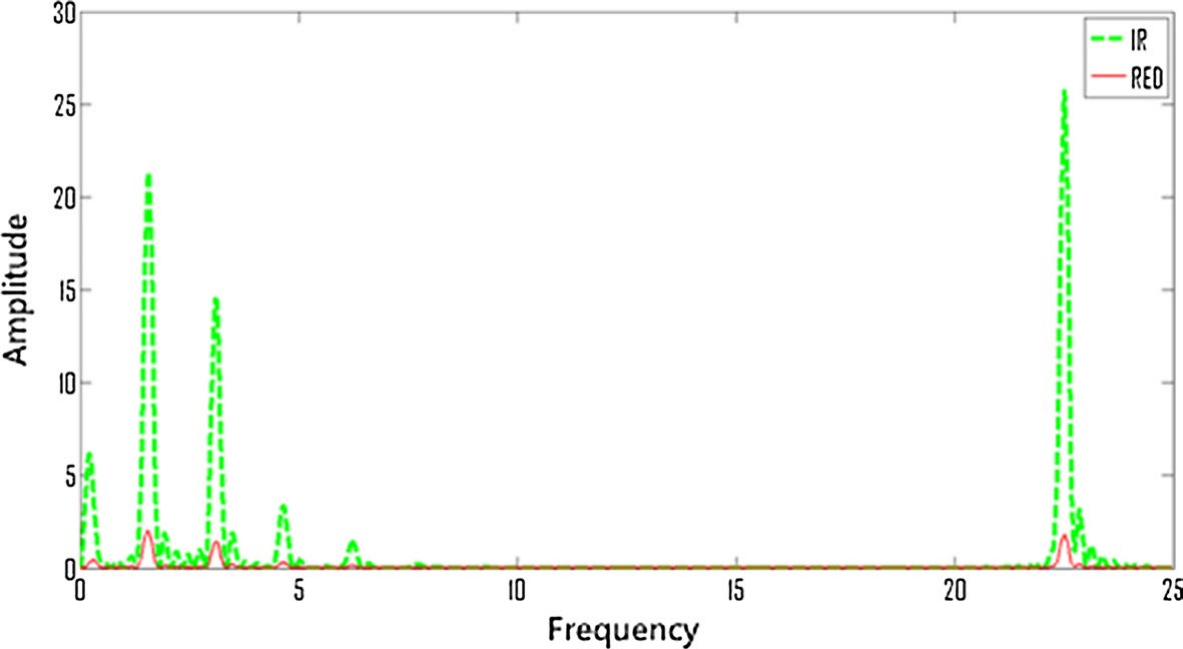
Respiration peak and pulse rate peak

In this manuscript, we introduced a system for the continuous and noninvasive measurement of SvO2. Our system utilizes a control system to modulate the adjustable stimulation signal that is essential to the measurement process, in which the stimulation signal rate shouldn’t be the same as patients’ pulse rate. The power spectrum of PPG in Fig. 3 can be found in Fig. 5, which can be divided into low frequency spectrum for pulse and high frequency spectrum for stimulation signal. The peaks of the power spectrum of the two kinds of signal are separated in frequency domain. In this way the ordinary FIR filter can be used to extract the pulse spectrum for the calculation of arterial oxygen saturation easily, and the stimulation signal for the calculation of the venous oxygen saturation. The experimental results indicate that the method of addition artificial stimulation can successfully transform the vein interference signal of pulse oxygen saturation measurement into useful signal to venous oxygen saturation measurements without compromising the pulse oxygen measurement. In addition, under hypoxic conditions, the system can reflect the overall decline of venous oxygen saturation better.

**Fig. 6:**
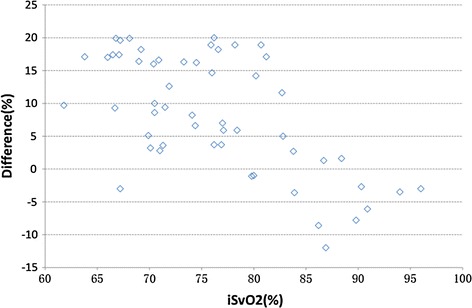
The difference of SvO2 between co-oximetry and noninvasive measurements

**Fig. 7:**
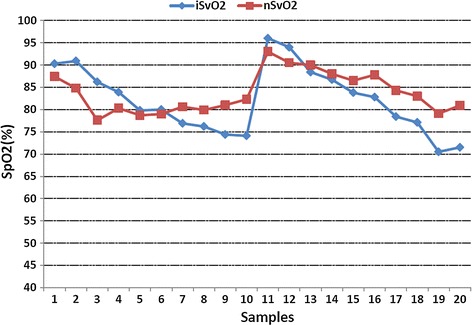
SvO2 trend chart of the new method system and co-oximetry

**Fig. 8:**
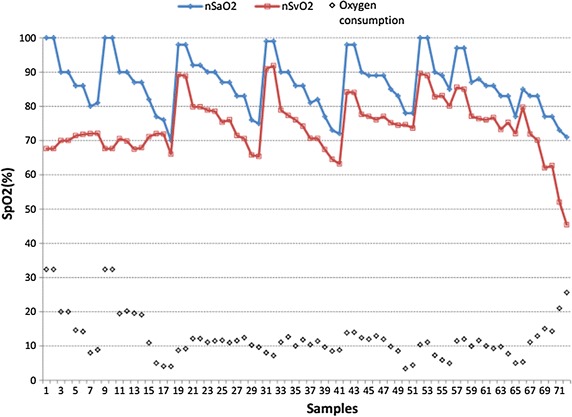
The oxygen consumption of body measured by noninvasive method

**Fig. 9:**
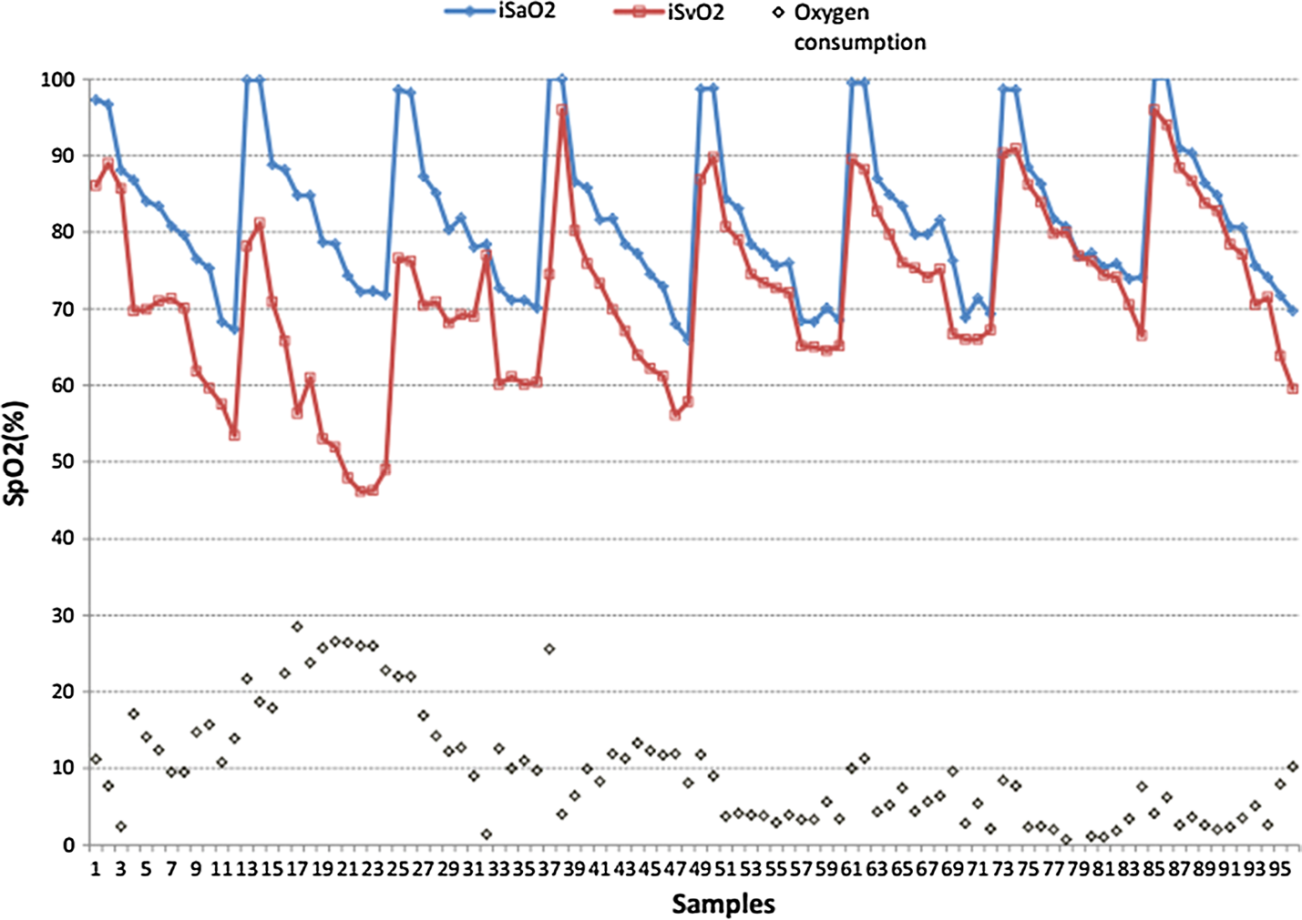
The oxygen consumption of body measured by invasive method

To identify the stability and reliability of the system, we studied nine healthy adults. Five volunteers took part in the invasive experiment and others took part in the noninvasive experiment. Invasive blood gas analysis (co-oximetry, golden criterion) was used to benchmark the accuracy and stability of the system. Referring to the standard calibration process of SpO2, samples of venous blood near from subjects’ fingers were collected. The sampling point is approximately set in 6 levels of oxygen saturation, and recorded twice for each subject (Fig. 4). The difference between the values which measured by the system and co-oximetry is almost within −10 to 10, as shown in Fig. 6, which includes all the data points without excluding any outliers. Figure 7 displays changes in nSvO2 (noninvasive SvO2) and iSvO2 (invasive SvO2) through different oxygen saturation levels from one subject. The root mean square error (the difference of SvO2-iSvO2) is 5.31 and the correlation coefficient (the difference of SvO2-iSvO2) is 0.72. In general, the system is stable but the accuracy should be further improved. Oxygen consumption is equal to SaO2 minus SvO2. Figure 8 shows is the oxygen consumption estimated by the noninvasive method, and Fig. 9 is the oxygen consumption estimated by the invasive method. The oxygen consumptions in the two figures are very close. Therefore, the new method can be used to assess the body oxygen consumption.

## Conclusions

The experimental results indicate that the noninvasive method can be a feasible approach for SvO2 measurement. Adding the external stimulation signal to vein solved the problems of the lack of fluctuation signal and improved signal-to-noise ratio in the measuring process. This new method of SvO2 measurement can potentially meet the unmet clinical needs, reducing the time and cost involved.

## Additional file

10.1186/s12938-016-0208-8 Raw Data.

## Abbreviations

SvO2: blood oxygen saturation of vein
SaO2: blood oxygen saturation of artery
PPG: photoplethysmography
nSvO2: noninvasive SvO2
iSvO2: invasive SvO2
